# Perceived Financial Strain and Adolescent Mental Health: Evidence from a Population-Based Study in South Tyrol, Italy

**DOI:** 10.3390/children13010121

**Published:** 2026-01-13

**Authors:** Christian J. Wiedermann, Verena Barbieri, Hendrik Reismann, Giuliano Piccoliori, Adolf Engl, Doris Hager von Strobele-Prainsack

**Affiliations:** 1 Institute of General Practice and Public Health, Claudiana College of Health Professions, 39100 Bolzano, Italy; 2 Faculty of Social Work, Health and Nursing, Ravensburg-Weingarten University of Applied Sciences (RWU), 88250 Weingarten, Germany; hendrik.reismann@rwu.de

**Keywords:** adolescent depression, adolescent anxiety, financial stress, socioeconomic stressors, perceived inflation, South Tyrol, mental health

## Abstract

**Highlights:**

**What are the main findings?**

- Self-perceived burden due to rising prices was the strongest socioeconomic correlate of adolescent mental health, showing consistent associations with depressive symptoms, emotional/behavioral difficulties and anxiety.
- Parental burden reports and objective family affluence showed weak or no associations, and female adolescents were particularly vulnerable to the psychological impact of financial strain.

**What are the implications of the main findings?**

- Subjective financial stress among adolescents should be prioritized in assessment and prevention, as it is a more proximal indicator of mental health risk than structural socioeconomic status measures.
- Public health strategies should directly address the psychological experience of financial strain, complementing economic policies with school-, family-, and community-based interventions, with particular attention to supporting the mental health of adolescent girls.

**Abstract:**

**Background/Objectives:** Socioeconomic stressors, such as financial strain, rising living costs, and perceived price burden, have gained relevance in the post-pandemic period and may adversely affect adolescent mental health. This study examined the association between subjective financial stress and symptoms of depression, anxiety, and emotional/behavioral difficulties among adolescents in Northern Italy. **Methods:** Data were obtained from the 2025 Corona and Psyche South Tyrol (COP-S) population survey. A total of 2554 adolescents aged 11–19 years and their parents participated; 1598 adolescents provided complete data for analyses of socioeconomic stressors (parent-reported Family Affluence Scale III, adolescent self-reported and parent proxy and self-reported burden due to price increases). Mental health outcomes included depressive symptoms (PHQ-2), generalized anxiety (SCARED-GAD), and emotional/behavioral difficulties (SDQ). Associations were assessed using chi-square tests, Kendall’s tau correlations, and two-factor ANOVA models. **Results:** Elevated depressive symptoms were present in 10.7% of adolescents, emotional/behavioral difficulties in 13.9%, and anxiety symptoms in 27.9% of adolescents. Female adolescents consistently showed higher symptom levels in all domains. Self-reported financial burden was the strongest and most consistent correlate of mental health problems, demonstrating small-to-moderate positive correlations with depressive symptoms (τ = 0.20, *p* < 0.001), emotional/behavioral difficulties (τ = 0.14, *p* < 0.001), and anxiety (τ = 0.25, *p* < 0.001). Parent-reported burden showed weaker and less consistent associations, and the Family Affluence Scale III was not significantly related to any of the mental health outcomes. ANOVA models indicated that adolescents’ own perception of financial burden significantly predicted anxiety levels in both age groups (11–14 and 15–19 years), whereas discrepancies between adolescent and parent burden perceptions were particularly relevant among younger adolescents. **Conclusions:** In this affluent European region, subjective financial strain, especially adolescents’ perception of burden due to rising prices, is a stronger determinant of depressive symptoms, anxiety, and psychosocial difficulties than parental burden reports or structural affluence indicators. Adolescents, especially females, appear to be particularly vulnerable. These findings underscore the importance of addressing subjective financial stress in adolescent mental health and public health strategies.

## 1. Introduction

Adolescent depression is a growing public health concern, with rising prevalence and early onset trajectories that can profoundly affect individual development and long-term societal outcomes [1]. It is associated with academic underachievement, social withdrawal, self-harm, and suicide, which is the second leading cause of death among adolescents [2].

While genetic, biological, and interpersonal factors contribute to adolescent mental health, there is increasing recognition of the role of broader structural stressors [3,4]. Socioeconomic living conditions, such as financial insecurity, housing strain, and the rising cost of living, are emerging as key environmental determinants of psychological well-being among youth [5]. These stressors may operate through both material deprivation and emotional pathways, including exposure to parental stress, reduced participation in social activities, and chronic instability in daily life [6,7,8,9]. Although dimensions such as financial hardship, insecure housing, and inflation-related stress have been studied in isolation, few studies have examined them jointly in representative youth samples [6,10].

Beyond financial strain, other socioeconomic stressors have been shown to contribute to adolescent vulnerability. A recent systematic review concluded that financial hardship is robustly associated with mental health impairments across developmental stages, including elevated depressive symptoms and internalizing behaviors in adolescents [11]. Housing instability has also been linked to poorer youth mental health, with insecure housing arrangements, frequent relocations, and overcrowding contributing to emotional dysregulation, social isolation, and perceived insecurity [12]. Longitudinal evidence suggests that housing-related stress may exert independent and lasting effects, even when controlling for socioeconomic status [13]. More recently, perceived inflation and cost-of-living pressures have emerged as novel forms of environmental stress. Although under-researched in pediatric populations, adolescents in various countries have identified economic worries as a dominant source of psychological stress, potentially affecting emotional well-being and social participation through heightened anxiety and vicarious exposure to caregiver stress [14].

Despite the growing attention to adolescent depression, there is a lack of integrated approaches that include perceived inflation, housing stress, and subjective financial strain within a single analytical framework [6,7,15]. Furthermore, most available studies focus exclusively on either self- or parent-reported data, often within limited age ranges. Comparative population-based studies using both perspectives across adolescence are needed to inform targeted public health responses [16,17,18].

In Italy, adolescent mental health has become a critical public health issue. Data show that depressive disorders affected 3.6% of adolescents aged 15–19 years in 2018, predominantly among females [19,20]. Surveys during and after COVID-19 revealed widespread anxiety, depression, and psychosocial difficulties among Italian youth [21,22]. Analyses indicate increasing psychological symptoms among Italian adolescents over the past decade, especially in teenage girls [23]. These findings highlight that Italian youth face significant mental health challenges, particularly amid economic and inflation-related pressures.

This study is anchored in the stress-process and social determinants frameworks, which assert that mental health outcomes are influenced by both objective socioeconomic conditions and individuals’ subjective evaluations of stressors and psychosocial contexts [24,25,26]. Structural indicators, such as family socioeconomic status, represent distal conditions, whereas perceived financial strain serves as a proximal stressor that directly impacts emotional functioning [27,28]. During adolescence, a developmental stage characterized by cognitive autonomy and heightened sensitivity to environmental stressors, subjective interpretations of economic insecurity are particularly significant [5]. Discrepancies between adolescent and parental perceptions of financial burden may indicate differences in stress appraisal, which contribute to mental health vulnerability [29]. This framework informed the selection of variables and the analytical focus on self- and proxy-reported socioeconomic stressors in relation to depressive symptoms, anxiety, and psychosocial difficulties.

This study used data from the “Corona and Psyche South Tyrol” (COP-S) 2025 population survey in Northern Italy to examine the associations between three key socioeconomic stressors (perceived adolescents’ burden of inflation, perceived parental burden of inflation, and socioeconomic status) and screening symptoms of depression, anxiety, and psychosocial difficulties in adolescents aged 11–19 years [30]. By including both adolescent self-reports and parent proxy assessments, this analysis aims to capture the complex relationship between environmental stress and youth mental health from two different familiar perspectives.

## 2. Methods

### 2.1. Study Design and Sample

South Tyrol was selected as the study setting due to its status as a high-income European region characterized by generally favorable living conditions. This context facilitates the investigation of socioeconomic stressors, such as perceived financial strain and inflation-related burden, in an environment where absolute material deprivation is relatively rare. This setting allows for a focused examination of subjective socioeconomic stress as a determinant of adolescent mental health, extending beyond traditional indicators of disadvantage. Recruitment through the public school system was employed to ensure comprehensive population coverage across municipalities, school types, and sociodemographic groups, aligning with the population-oriented design of the COP-S survey series.

This cross-sectional study was based on the fourth wave of the COP-S survey series, which was conducted from March to April 2025. The anonymous online survey targeted families with school-aged children in South Tyrol in Northern Italy. Recruitment occurred through school directorates, who distributed invitations and access links via email to more than 40,000 families in the region. The survey was administered using the SoSci Survey platform (Version 3.2.46; SoSci Survey GmbH, Munich, Germany).

The 2025 wave retained the core structure of previous COP-S surveys (2021–2023) [21,30,31,32], building on the methodology of the German Corona and Psyche (COPSY) study [33], while incorporating additional items related to post-pandemic socioeconomic stress. The aim of the 2025 survey is to investigate the contemporary determinants of youth mental health, including perceived inflation.

For this analysis, only adolescents aged 11–19 years and their corresponding proxy reports were included in the study. Parental informed consent and adolescent assent were obtained electronically before participation. Adolescents completed a self-report questionnaire after their parents completed a proxy version. For stratified analyses, adolescents were grouped into two age categories reflecting early and middle-to-late adolescence (11–14 years and 15–19 years, respectively).

### 2.2. Measures

#### 2.2.1. Sociodemographic and Family Variables

Proxy-reported sex and age of children and adolescents were analyzed. Parental education was assessed using the CASMIN classification [34], and responses were categorized as low (primary/lower secondary), medium (upper secondary), or high (tertiary education). Migration background was defined as “yes” if at least one of the parents was not born in Italy. Parental mental health diagnosis was assessed using a binary self-report item. Physical activity was assessed via adolescent self-report, asking how many days in the past week they engaged in at least 60 min of sports or movement, with response options ranging from 0 to 7 days. Hours of media use were assessed separately for school-related and private purposes. Adolescents reported their average daily use in hours on a scale from 0, 0.5, 1, 2, 3, 4, and 5+ hours. Family structure was recorded based on whether the adolescent lived in a single-parent household or with two parents or caregivers. Urban vs. rural residence was recorded based on the municipality and classified as urban according to the official urban municipalities. Family language was assessed using German, Italian and Others, including Ladin, as categories.

#### 2.2.2. Socioeconomic Stressors

Perceived inflation was assessed as part of a broader battery of global crises, asking respondents how much they felt burdened by price increases. Responses were collected on a 5-point scale (1 = “not at all” to 5 = “very strong”). For ANOVA and dichotomized analyses, responses were grouped into ‘no burden’ (not at all/slight) and ‘burdened’ (moderate/strong/very strong).

Socioeconomic status (SES) was assessed using the Family Affluence Scale (FAS III), an established measure designed to capture material wealth in families. The FAS III is particularly suitable for youth research, where direct information on household income or parental occupation may be less reliable or difficult to collect [35,36,37,38,39].

FAS III was operationalized based on parent responses to the following six items, which were validated for international comparisons [39,40]:

- Car ownership: “Does your family own a car?” The response options were coded as 0 (no), 1 (one car), and 2 (two or more cars).
- Own bedroom: “Does your child have its own room?” (yes/no).
- Number of computers: Total number of computers, laptops, or tablets in the household (excluding gaming consoles and smartphones), coded from 0 to 3+.
- Number of bathrooms: Total number of bathrooms in the household, coded from 0 to 3+.
- Dishwasher ownership: Presence of a dishwasher in the household (yes/no).
- Holiday frequency: Number of family vacations taken in the past year, with response options ranging from 0 (none) to 3 (more than twice per year).

These items were aggregated to create a composite FAS III, reflecting material affluence, with higher scores indicating greater family wealth. The FAS has demonstrated good external validity, correlating with traditional SES markers such as parental education and regional income [37,38,41], and health outcomes in adolescents [36,42]. It achieves completion rates exceeding 95%, making it a robust tool for large-scale population studies [36,43].

For the analysis, the FAS III score was considered a continuous variable. Tertiles of FAS scores were constructed and categorized into groups according to [44]. In the present sample, this corresponded to FAS III scores of 1–7 for low affluence (20.3%), 8–10 for medium affluence (54.5%), and 11–13 for high affluence (25.2%).

#### 2.2.3. Mental Health Outcomes

Three validated screening tools were used to assess adolescent mental health, all of which had been applied identically in previous COP-S surveys with detailed documentation of their structure, psychometric properties, and categorization criteria [32].

- PHQ-2 (Patient Health Questionnaire-2): A 2-item screener for depressive symptoms, with a score of ≥ 3 indicating elevated symptoms [45,46].
- SCARED-GAD (Generalized Anxiety Subscale): The 9-item generalized anxiety subscale of the Screen for Child Anxiety-Related Emotional Disorders. A score of ≥ 9 was used to classify elevated anxiety symptoms [47,48].
- Strengths and Difficulties Questionnaire (SDQ): The self-rated SDQ was used to evaluate emotional and behavioral difficulties [49]. For this study, the Total Difficulties Score (excluding the prosocial scale) was dichotomized based on official cut-offs, with “borderline” and “abnormal” classifications grouped as elevated.

The measures employed in this study are conceptually related yet operationally distinct. Socioeconomic stressors were evaluated independently from mental health outcomes, ensuring no overlap between exposure and outcome measures. The PHQ-2, SCARED-GAD, and SDQ instruments assess different dimensions of adolescent mental health without shared items; thus, the associations observed reflect symptom co-occurrence rather than measurement overlap. Although comorbidity between symptoms is anticipated during adolescence, each instrument captures a distinct construct and was analyzed separately.

### 2.3. Statistical Analysis

Descriptive statistics were calculated for all variables. Continuous variables are summarized as mean ± standard deviation (SD), whereas categorical variables are reported as frequencies and percentages. Confidence intervals (CI) for proportions were calculated using binomial methods. Differences in categorical variables were tested using chi-square tests, and ordinal and non-normally distributed metric variables were compared using the Mann–Whitney U test.

The primary outcomes (depressive symptoms, anxiety symptoms, and elevated SDQ scores) were analyzed as binary variables. Associations with perceived inflation were first explored using unadjusted odds ratios (ORs).

To examine bivariate associations between socioeconomic stressors (FAS III, self- and parent-reported price burden) and continuous symptom scores (PHQ-2, SCARED-GAD, SDQ total), Kendall’s tau-b correlations (two-tailed) were computed with pairwise deletion of the missing data. Kendall’s tau-b was chosen due to the ordinal nature of the stressor variables and the presence of tied ranks. Effect size interpretation for correlation coefficients followed the empirically derived guidelines by Gignac and Szodorai, where τ ≈ 0.10 is considered small, τ ≈ 0.20 is typical/medium, and τ ≥ 0.30 is relatively large [50].

To examine whether different patterns of perceived financial burden were associated with anxiety in adolescents, we performed two-factor ANOVA models with interaction terms stratified by age group, as defined above. Each model included two binary factors (e.g., adolescent self-reported burden: yes/no; parental proxy-reported burden: yes/no) and their interactions. Homogeneity of variance was assessed using Levene’s test. For each model, effect sizes were quantified using partial eta squared (ηp^2^), derived from the ANOVA tests. Model-adjusted marginal means were estimated and plotted to visualize the significant main and interaction effects.

All analyses were conducted using IBM SPSS Statistics for Windows (version 25.0; IBM Corp., Armonk, NY, USA). Statistical significance was set at *p* < 0.05.

### 2.4. Use of Generative Artificial Intelligence (GenAI)

GenAI (ChatGPT, OpenAI) was used only in a limited and auxiliary manner during manuscript preparation, namely to support the structuring and linguistic refinement of individual text passages. AI was also used to synthesize and cross-reference the existing literature for clarity and contextualization. No generative AI was used for data collection, statistical analysis, or interpretation of the results. All the content was reviewed and approved by the authors.

## 3. Results

### 3.1. Sample Characteristics

A total of 2554 adolescents aged 11–19 years participated in the survey. Of these, 1598 adolescents had complete data for the FAS III and self-reported burden due to price increases and were included in the main analysis. Of the 1598 adolescents included in the analysis, 826 (51.7%) were aged 11–14 years and 772 (48.3%) were aged 15–19 years. The mean age of the participants was 14.4 years (SD 2.3), and gender was evenly distributed (50.0% male, 49.94% female, 0.06% diverse). Most adolescents lived in two-parent households (88.1%), and the majority resided in rural areas (71.3%). A migration background was reported by 8.5% of adolescents. Parents were predominantly in the high (41.8%) or medium (39.2%) educational categories, and 4.5% of families reported a current parental psychological or psychosomatic burden. German was the most common family language (82.2%), followed by Italian (14.1%) and Ladin/other languages (3.7%).

Regarding socioeconomic conditions, 23.1% of adolescents reported being highly burdened by rising prices, whereas only 10.8% of parents perceived their adolescents as highly burdened. In contrast, 59.4% of parents reported a high own burden due to price increases. According to the FAS III, 25.9% of adolescents were categorized as having high affluence, 56.9% as having medium affluence, and 17.2% as having low affluence.

In terms of health-related behaviors, adolescents engaged in physical activity on average 3.5 ± 1.9 days per week, reported 2.4 ± 1.3 h of daily private screen time, and 1.2 ± 1.2 h of screen time for school.

Comparisons between younger (11–14 years) and older (15–19 years) adolescents revealed no significant differences in the demographic characteristics. However, age groups differed in lifestyle behaviors: younger adolescents reported more physical activity (3.89 vs. 3.14 days/week, *p* < 0.001) and less screen time for both school purposes (0.88 vs. 1.79 h/day, *p* < 0.001) and private use (2.10 vs. 2.72 h/day, *p* < 0.001) than older adolescents. The indicators of socioeconomic stress showed mixed patterns. Both adolescents and parents reported greater price-related burden among older adolescents (*p* = 0.005 for both), whereas FAS III scores and parents perceived financial burden did not differ by age group.

According to previously published findings from the COP-S 2025 dataset [30], mental health symptoms were common, with 27.9% screening positive for anxiety (SCARED), 13.9% for general emotional/behavioral problems (SDQ), and 10.7% for depressive symptoms (PHQ-2), with a higher prevalence among females and adolescents living in single-parent households. No associations with parental education or migration background were observed.

### 3.2. Distribution of Socioeconomic Stressors

Self-reports of adolescents indicated that approximately half perceived at least a moderate burden from price increases, and one in four rated the burden as strong or very strong (Table 1). Perceived burden was significantly higher among older than younger adolescents (*p* < 0.001). In contrast, parental reports showed a more favorable distribution; more than two-thirds rated their child’s burden as not at all or only slightly pronounced, and 10.7% reported a strong or very-strong burden. Parents of older adolescents also perceived their children’s burden to be significantly more severe (*p* < 0.001).

Parental self-perceived burden was very high overall, with approximately 90% reporting at least a moderate burden, and it did not differ between the two age groups. Table 1 summarizes these findings.

The associations between the informants ranged from moderate to large. The correlation between adolescent self- and parent-reported burden was τ = 0.300 (*p* < 0.001; medium effect size). The association between parent-reported child burden and parental self-burden was τ = 0.525 (*p* < 0.001; large effect size). The correlation between adolescent and parental self-burden was τ = 0.385 (*p* < 0.001; medium-to-large effect).

As shown in Table 2, the FAS III indicators reflect generally high levels of material affluence among the families included in the study. According to parental reports, only 1.4% of households did not own a car, whereas 61% owned two or more. A large majority of adolescents (86.2%) were reported to have their own bedroom, and two-thirds lived in households with three or more computers. Household amenities were similarly common, with more than half of families reporting at least two bathrooms and 94.6% reporting the presence of a dishwasher. In terms of family holidays, almost nine in ten families had taken at least one trip in the previous year, and 22.7% had travelled more than twice.

Associations between FAS III score and perceived burden due to price increases were statistically significant but small in magnitude. Higher family affluence showed a small negative correlation with the self-reported burden of adolescents (τ = –0.093, *p* < 0.001), indicating that more affluent adolescents perceived slightly less strain from rising prices. A similar small effect was observed for parental proxy reports of the burden of adolescents (τ = –0.148, *p* < 0.001). The strongest association emerged for parental self-perceived burden (τ = –0.266, *p* < 0.001), although this still fell within the upper small-to small-to-medium effect-size range.

### 3.3. Economic Stressors and Demographic Parameters

As shown in Table 3, sex, urban residence, parental mental health problems, and physical activity were not associated with any of the economic stress indicators. In single-parent households, parent-perceived adolescent burden and parental self-burden were significantly higher in both age groups, and low FAS III scores were substantially more common. Among younger adolescents, self-reported burden was significantly higher in single-parent families. For younger adolescents with a migration background, parental self-burden was significantly lower than that for families without a migration background.

Higher parental education was consistently associated with lower levels of burden across all indicators, except for proxy-reported adolescent burden in younger age groups. With respect to family language, older adolescents from households not speaking German or Italian at home showed significantly higher self- and proxy-rated burdens.

Self-perceived burden was significantly higher among younger adolescents who reported more than two hours of private screen time per day and among older adolescents who used more than two hours of school-related screen time. Finally, younger adolescents who spent more than two hours per day on school-related screen activities were significantly more likely to fall into the low FAS III category.

### 3.4. Associations Between Socioeconomic Stressors and Mental Health Outcomes

As shown in Table 4, socioeconomic stressors were consistently associated with poorer adolescent mental health outcomes. Across all three measures, parental self-reported burden due to price increases showed the strongest and most consistent associations, with significant positive correlations with depressive symptoms, anxiety, and emotional/behavioral difficulties in the total sample as well as in both sexes and age groups. Effect sizes were modest but stable and tended to be slightly stronger among females.

Adolescents’ self-reported burden due to price increases was consistently associated with anxiety symptoms across the total sample, sexes, and age groups, but showed weaker and less consistent associations with depressive symptoms and emotional or behavioral difficulties. Associations with depressive symptoms were significant primarily in females and younger adolescents, whereas associations with SDQ scores were limited to the total sample. Parent-reported adolescent burden demonstrated modest but significant associations with anxiety symptoms across sexes and age groups, while associations with depressive symptoms and emotional or behavioral difficulties were limited and inconsistent, reaching significance only in selected subgroups.

Family affluence was not significantly related to any mental health outcome in the total sample. The only exceptions were a very small positive association between higher family affluence and anxiety symptoms among male adolescents and there is a small negative association with SDQ among females.

### 3.5. Associations Between Financial Burden and Anxiety (ANOVA Models)

**Figure 1:**
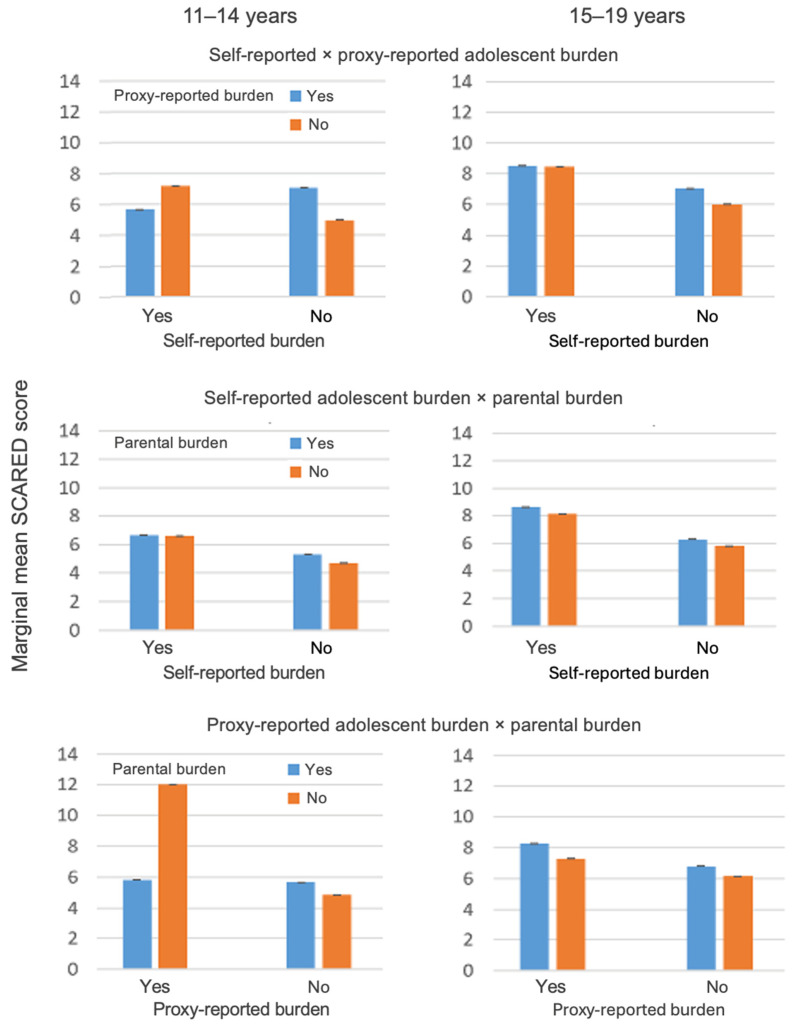
Marginal means of adolescent anxiety symptoms (SCARED total score) by combinations of self-, proxy, and parental financial burden, stratified by age group.

Table 5 summarizes the two-factor ANOVA models examining the effects of perceived financial burden on adolescent anxiety (SCARED-GAD total score) separately for the two age groups. Figure 1 presents the model-adjusted marginal means for each burden combination.

#### 3.5.1. Self- and Proxy-Reported Adolescent Burden

Among 15–19-year-olds, only proxy-reported adolescent burden was significantly associated with higher SCARED scores, whereas adolescents’ self-reported burden was not. In contrast, among 11–14-year-olds, the interaction between self- and proxy-reported burden was the only significant effect. As visualized in Figure 1, younger adolescents showed the highest anxiety levels when adolescents and parents disagreed in their perception of financial burden.

#### 3.5.2. Self-Reported Adolescent Burden vs. Parental Self-Burden

In both age groups, adolescents’ self-perceived financial burden remained the only consistently significant predictor of anxiety symptoms. Parental self-burden and the interaction term did not reach significance in either age group. As shown in Table 5 and Figure 1, higher self-reported burden was associated with higher SCARED scores among both younger and older adolescents.

Although none of the other predictors were significant, the overall models were significant in both age groups (*p* < 0.001), indicating that perceived financial burden, as captured primarily by adolescents’ own appraisals, explains a statistically significant, albeit modest, proportion of anxiety symptoms.

#### 3.5.3. Proxy-Reported Adolescent Burden vs. Parental Self-Burden

Among 15–19-year-olds, neither proxy-reported adolescent burden nor parental self-burden, nor their interaction, was significantly associated with anxiety symptoms, although the overall model reached statistical significance (*p* = 0.006; Table 5). In contrast, among 11–14-year-olds, the interaction between proxy-reported adolescent burden and parental self-burden was statistically significant (ηp^2^ = 0.006, *p* = 0.025), while the individual main effects were not. As illustrated in Figure 1, anxiety levels among younger adolescents were highest in constellations where parental perceptions of the child’s burden and parents’ own perceived financial burden diverged. Including single-parent household as an additional fixed factor did not alter the pattern of results and is therefore not shown.

## 4. Discussion

This population-based study examined the role of socioeconomic stressors, particularly perceived financial strain related to inflation, in adolescent mental health outcomes, including depressive symptoms, anxiety, and emotional or behavioral difficulties. Overall, perceived financial burden was significantly associated with poorer mental health, although the strength and consistency of associations varied by outcome and informant. Anxiety symptoms demonstrated the strongest and most consistent relationships with financial stress across sexes and age groups, with effect sizes exceeding those observed for depression and emotional challenges. This suggests that anxiety may represent the mental health domain most immediately responsive to acute socioeconomic pressures such as perceived inflation and family financial strain, whereas depressive symptoms and emotional difficulties may reflect more cumulative or indirect pathways of influence. Together, these findings reinforce the importance of socioeconomic stress in shaping adolescent mental health, while highlighting differential sensitivity across symptom domains.

### 4.1. Socioeconomic Stress and Adolescent Mental Health

The findings of this survey confirm that subjective financial strain, particularly adolescents’ own perception of burden due to rising prices, is more closely associated with mental health symptoms than structural indicators of socioeconomic status, such as FAS III. Subjective financial stress was strongly associated with adolescent mental health, with adolescents’ self-reported burden showing particularly consistent associations with anxiety symptoms, while parental self-reported burden emerged as the most consistent correlate across all mental health outcomes. This aligns with evidence demonstrating that perceived financial hardship is a more immediate and salient psychological stressor than objective material resources [7,51,52]. Earlier studies have also shown that financial worries and the emotional experience of economic insecurity are more predictive of internalizing symptoms than income or assets, which tend to influence mental health indirectly via stress and coping pathways [53,54,55]. Mechanisms proposed in the literature, including heightened psychological stress, reduced social participation, vicarious exposure to parental worries, and uncertainty about financial stability, are consistent with the patterns observed in our data [7,54,56]. Overall, the results reinforce the broad evidence from diverse populations indicating that subjective financial strain is a robust correlate of mental health across developmental stages and socioeconomic contexts [57,58,59,60].

Psychological distress during adolescence cannot be comprehended solely as a reaction to external stressors. This developmental stage is marked by normative intrapersonal processes, such as identity formation, self-affirmation, and increased emotional reactivity, through which distressing experiences may contribute to development [61]. In this context, the significant associations observed with adolescents’ subjective financial strain likely reflect not only exposure to socioeconomic stress but also the interpretation and integration of such stressors within ongoing developmental processes. Even in favorable socioeconomic conditions, adolescents may experience distress as part of normative development, highlighting that subjective vulnerability does not imply pathology but rather reflects the interaction between environmental challenges and intrapersonal maturation.

Parental reports of the price-related burden on adolescents showed weaker and less consistent associations with mental health symptoms, reaching significance mainly for anxiety and only in specific subgroups. This divergence highlights the unique importance of the subjective appraisal of financial stress among adolescents, which was particularly salient for anxiety symptoms and more weakly associated with other mental health domains. Prior research similarly shows that adolescents’ perceptions of financial strain more robustly predict depressive symptoms, emotional distress, and even suicidal ideation than do parental assessments [62,63,64]. Developmentally, adolescents may interpret financial insecurity in ways that are more closely tied to their emotional state, particularly during a period characterized by increasing autonomy, growing economic awareness, and heightened sensitivity to family stressors [65]. In contrast, parental financial strain generally influences youth more indirectly through pathways such as parental distress, reduced emotional availability, or increased family conflict [66].

A further consideration pertains to the increasing discrepancy in perceptions of financial strain between adolescents and parents as the overall burden escalates. Such divergence may indicate underlying family communication and coping challenges, including parental overwhelm or the inclination to downplay or shield adolescents from stress, which can inadvertently contribute to feelings of isolation or misunderstanding among young individuals. These dynamics may exacerbate internalizing symptoms and, in some instances, serve as early indicators of more severe psychosocial distress. Furthermore, financial stress may manifest not only in internalizing domains but also in irritability, impulsive or aggressive behavior, social withdrawal, school disengagement, and sleep disturbances. These discrepancy effects were observed primarily among younger adolescents, whereas no comparable interaction patterns were evident in older adolescents.

The favorable distribution in parental reports highlights significant differences in perspective between parents and adolescents [29]. Parents tend to evaluate their child’s burden based on material security and protective intentions, often minimizing stress to preserve their sense of parental efficacy. In contrast, adolescents assess financial strain through emotional experiences, social comparisons, and perceived constraints. These distinct perspectives elucidate why parental assessments indicate a lower burden, whereas adolescents’ appraisals exhibit a stronger correlation with mental health symptoms.

The discrepancies between adolescents’ and parents’ socioeconomic evaluations highlight the limitations of survey data in capturing the subjective aspects of financial stress. While adolescents’ self-reported financial burdens are strongly correlated with depressive symptoms, parental perceptions may reflect differing priorities or protective intentions. Employing qualitative methods, such as interviews, could elucidate how adolescents experience economic strain, how financial concerns are communicated within families, and why there are differences in burden assessments between parents and children.

The FAS III, a validated and widely used indicator of material resources in adolescent health research [35], showed no significant association with depressive symptoms, anxiety, or emotional/behavioral difficulties in this study’s findings. This is consistent with critiques that traditional structural SES indicators often underestimate the psychosocial dimensions of socioeconomic stress, particularly in comparatively affluent regions, where variability in material conditions is limited [67,68]. In such contexts, adolescents may experience financial strain less through material deprivation and more through subjective perceptions of rising costs, relative social standing, or family economic uncertainty—factors that have been identified as stronger predictors of adolescent well-being than objective affluence [69,70].

### 4.2. Gender Differences

Although the associations between socioeconomic stressors and depressive symptoms were weaker than those with anxiety or emotional/behavioral difficulties, our findings still showed that depression was more prevalent among female adolescents, consistent with prior research. Female adolescents in our study also reported substantially higher anxiety and emotional or behavioral difficulties than males. This pattern is consistent with previous research documenting a higher prevalence of internalizing symptoms among adolescent girls [71,72,73].

The gender gap in internalizing symptoms typically widens during adolescence, reflecting girls’ heightened sensitivity to psychosocial stress, greater reliance on ruminative and emotion-focused coping strategies, and the influence of gendered social expectations that may amplify emotional strain [74,75]. Consistent with these mechanisms, our results showed that the association between perceived burden due to rising prices and mental health symptoms was stronger among female adolescents. Similar patterns have been documented in recent studies, where socioeconomic stressors—especially financial strain—exert more pronounced psychological effects on girls than on boys [76,77]. Together, these findings suggest that adolescent girls may be particularly vulnerable to the mental health consequences of financial stress, likely due to a combination of heightened psychosocial reactivity, stress-amplifying coping styles and sociocultural pressures.

### 4.3. Broader Context and Implications

In recent European surveys, economic worries and perceived inflation have ranked among the most frequently cited stressors reported by adolescents and families [78,79,80]. Our findings reinforce that inflation is not merely a macroeconomic phenomenon but also a psychosocial stressor with measurable implications for youth’s mental health. Importantly, it is the subjective experience of rising prices—worrying about daily living costs and financial instability—rather than objective hardship alone that is most strongly associated with anxiety, emotional difficulties, and, to a lesser extent, depressive symptoms [78,79,81]. The COVID-19 pandemic further intensified socioeconomic disparities and created new vulnerabilities for young people, with family level financial insecurity emerging as a stronger predictor of stress than school closures [82].

Together, these findings highlight the importance of addressing household-level socioeconomic stress as a determinant of adolescent well-being in a post-pandemic context marked by inflation, housing shortages, and increasing income inequality. Therefore, public health strategies should move beyond income redistribution alone and address the psychological dimensions of financial strain. School-based mental health programs [83], community counseling, and family oriented prevention initiatives should integrate financial stress as a core theme while simultaneously promoting resilience and reducing the stigma surrounding economic worries [78,84]. Given the increasing digitalization of social support systems and the persistent division of responsibilities between schools and youth work, adolescents’ experiences of financial stress need to be understood within the broader context of their rapidly changing social and digital environments, which may shape both their exposure to stressors and access to coping resources [85,86].

The findings of this study have significant theoretical and empirical implications. Theoretically, they lend support to stress-process and social determinants frameworks by demonstrating that adolescents’ subjective perceptions of socioeconomic stress are more closely linked to mental health symptoms than are structural indicators of family affluence. This highlights the necessity of incorporating perceived financial strain and discrepancies in parent-adolescent perceptions into models of adolescent mental health vulnerability.

Empirically, the results underscore the importance of employing multi-informant designs that integrate both subjective and objective socioeconomic measures. Future research should employ longitudinal and mixed methods approaches to elucidate temporal pathways and to gain a deeper understanding of how adolescents experience and interpret financial stress in their daily lives.

### 4.4. Strengths and Limitations

This study had several strengths. First, it is based on a large population-level dataset covering adolescents aged 11–19 years in a bilingual European region, enabling age- and gender-stratified analyses with adequate statistical power. Second, the simultaneous use of adolescent self-reports and parent proxy reports provides a more nuanced understanding of socioeconomic stress than single-informant studies. Third, validated mental health screeners (PHQ-2, SCARED-GAD, SDQ) were used, ensuring comparability with the international literature. Importantly, this study not only confirms prior evidence that subjective financial strain is more closely related to adolescent mental health than objective material affluence, but also adds novel contributions by contrasting adolescent and parental perceptions of financial burden in the specific post-pandemic context of inflation and by demonstrating that structural indicators such as FAS III contribute only marginally to explaining mental health variation in a relatively affluent region such as South Tyrol.

This study has several limitations. First, the cross-sectional design prevents causal inference. It remains unclear whether financial stress increases mental health symptoms, whether adolescents with emotional or anxiety vulnerabilities perceive financial strain more acutely, or whether both processes operate simultaneously through a bidirectional reinforcement. Although the ANOVA models provide insight into how different combinations of parent- and adolescent-perceived burden relate to variations in anxiety levels, ANOVA does not establish temporal ordering or causality; it merely quantifies group differences in current symptoms levels. Therefore, while the observed associations are consistent with the theoretical mechanisms linking financial strain to poorer mental health, alternative explanations cannot be excluded.

Second, all socioeconomic stressors and mental health outcomes were based on self- or proxy-reported data without clinical diagnostic verifications. This may introduce reporting bias or reflect differences in perceptual thresholds, rather than true differences in symptomatology. Third, because the response rate could not be precisely calculated, selection bias was possible. The skewed distribution of FAS III, particularly the unexpectedly high frequency of multiple bathrooms, suggests that more affluent households may be overrepresented. Nevertheless, retaining the FAS III was important for comparability with international adolescent health studies and for providing a structural anchor against which subjective financial strain could be contrasted.

Fourth, missing data were substantial for several adolescent-reported variables, potentially reducing the precision of subgroup analyses. Fifth, some relevant socioeconomic indicators, such as detailed household income, debt, employment instability, and housing conditions, were unavailable. This limited the breadth of socioeconomic stressors that could be examined and prevented the integration of additional structural determinants. Finally, the study was conducted in the context of generally high living standards; therefore, the results may be generalized more readily to other affluent regions than to areas with greater material deprivation.

## 5. Conclusions

This population-based study from a high-income European region demonstrated that socioeconomic stress is closely linked to key dimensions of adolescent mental health. Depressive symptoms were present in approximately one in seven adolescents, emotional or behavioral difficulties in one in six, and anxiety in one in three. Among the stressors examined, perceived financial burden was a meaningful correlate of adolescent mental health, with adolescents’ own appraisals showing particularly robust associations with anxiety symptoms, while parental self-reported burden was more consistently related to depressive symptoms and emotional or behavioral difficulties. Although associations with anxiety and emotional/behavioral difficulties were evident, the relationship with depression was robust and clearly aligned with the study hypothesis. Female adolescents were particularly vulnerable, exhibiting higher rates of internalizing problems and stronger associations between financial strain and depression symptoms. These findings highlight that subjective socioeconomic stress functions as a proximal and psychologically salient determinant of adolescent depression, even in an affluent region where material deprivation is uncommon.

In the post-pandemic context characterized by inflation, rising living costs, and increased household financial insecurity, these results underscore the need for public health strategies that extend beyond structural indicators of socioeconomic status. Effective prevention should incorporate school-based, community, and family focused programs that address both material realities and the psychological burden of financial strain. By acknowledging and targeting subjective economic stressors, interventions can more effectively mitigate depressive symptoms and support the mental health of adolescents navigating a rapidly changing socio-economic landscape.

## Figures and Tables

**Table 1:** Prevalence and severity of perceived burden due to price increases among adolescents and parents, overall, and by age group.

Burden Due to Price Increases	Age Group	Valid n	Burden Rating (%)					*p*-Value
			Not at All	Slight	Moderate	Strong	Very Strong	
Adolescent (self-report)	11–19	1598	24.8	25.6	26.5	15.5	7.6	—
	11–14	826	30.3	24.6	24.9	14.2	6.1	<0.001
	15–19	772	19.0	26.7	28.1	17.0	9.2	
Adolescent (parental report)	11–19	1597	32.6	35.9	20.7	7.8	3.0	—
	11–14	820	38.2	34.5	18.7	6.0	2.7	<0.001
	15–19	767	26.6	37.4	22.9	9.6	3.4	
Parental (parental report)	11–19	1595	1.6	10.6	28.4	36.2	23.2	—
	11–14	824	1.8	9.0	28.0	37.3	23.9	n.s.
	15–19	771	1.3	12.3	28.8	35.1	22.4	

The percentages were based on valid responses. The *p*-values refer to the χ^2^ tests comparing the 11–14 and 15–19 age groups for each question. n.s. = not significant.

**Table 2:** Family Affluence Scale (FAS III) indicators among adolescents in South Tyrol.

FAS III Indicator	Valid n	Category	n (%)
Car ownership	1598	0 cars	22 (1.4)
		1 car	602 (37.7)
		2+ cars	974 (61.0)
Own bedroom	1598	Yes	1377 (86.2)
		No	221 (13.8)
Computers	1598	0	2 (0.1)
		1	170 (10.6)
		2	383 (24.0)
		3+	1043 (65.3)
Bathrooms	1598	0	633 (39.6)
		1	805 (50.4)
		2	160 (10.0)
		3+	213 (11.2)
Dishwasher	1598	Yes	1512 (94.6)
		No	86 (5.4)
Family holidays (last year)	1598	None	177 (11.1)
		Once	612 (38.3)
		Twice	446 (27.9)
		>2 times	363 (22.7)

**Table 3:** Sociodemographic and socioeconomic characteristics of adolescents in South Tyrol, stratified by age group.

Variable	Burden Due to Price Increases (%)						FAS III (Low) ^1^	
	Adolescent (Self-Report)		Adolescent (Parent-Report)		Parental (Parent-Report)			
	Age Group		Age Group		Age Group		Age Group	
	11–14	15–19	11–14	15–19	11–14	15–19	11–14	15–19
Gender								
Male	21.9	27.1	9.1	13.7	62.3	59.2	16.5	13.8
Female	18.4	25.3	8.2	12.4	60.0	56.1	20.1	18.2
*p*-value	n.s.	n.s.	n.s.	n.s.	n.s.	n.s.	n.s.	n.s.
Parental household								
Two-parent	19.2	25.7	7.8	12.1	58.6	54.7	14.5	12.0
Single parent	29.1	29.8	16.7	19.2	81.4	76.9	50.0	42.3
*p*-value	0.03	n.s.	0.006	0.046	<0.001	<0.001	<0.001	<0.001
Residence								
Urban	22.0	23.9	7.8	9.3	64.8	52.2	21.6	16.8
Rural	19.5	27.1	9.0	14.6	59.8	59.8	17.0	15.8
*p*-value	n.s.	n.s.	n.s.	n.s.	n.s.	n.s.	n.s.	n.s.
Migration background								
Yes	16.7	26.5	6.1	13.4	48.5	55.9	13.6	22.1
No	20.4	26.0	8.8	12.8	61.7	57.1	18.6	15.4
*p*-value	n.s.	n.s.	n.s.	n.s.	0.035	n.s.	n.s.	n.s.
Parental education								
Low	28.7	27.8	13.4	16.7	71.1	73.3	32.0	25.2
Medium	18.4	29.5	7.3	15.5	61.4	58.4	19.6	18.1
High	18.3	20.8	8.1	7.8	56.7	48.2	10.9	8.1
*p*-value	0.018	0.040	n.s.	0.006	0.010	<0.001	<0.001	<0.001
Parental mental problems								
No	19.7	26.1	8.3	13.0	61.3	56.7	17.9	15.6
Yes	28.9	29.4	13.2	14.7	57.9	73.5	23.7	20.6
*p*-value	n.s.	n.s.	n.s.	n.s.	n.s.	n.s.	n.s.	n.s.
Family language								
German	20.3	24.6	8.7	13.8	59.6	57.7	18.1	15.2
Italian	22.2	29.6	8.6	5.60	70.9	52.8	19.7	19.4
Other	11.1	43.6	7.4	22.6	55.6	71.9	18.5	18.8
*p*-value	n.s.	0.036	n.s.	0.018	n.s.	n.s.	n.s.	n.s.
Physical activity								
0–2 days a week	22.4	26.7	11.9	13.8	65.1	57.0	21.9	15.6
3 or more days a week	19.6	25.8	7,70	12.6	60.0	57.7	17.2	16.2
*p*-value	n.s.	n.s.	n.s.	n.s.	n.s.	n.s.	n.s.	n.s.
Screen time for private purposes								
0–2 h a day	17.6	26.4	7.7	14.8	60.6	57.1	17.4	16.6
2 or more hours a day	26.3	26.1	11.1	11.7	63.5	58.5	20.4	15.2
*p*-value	0.004	n.s.	n.s.	n.s.	n.s.	n.s.	n.s.	n.s.
Screen time for school purposes								
0–2 h a day	19.8	24.3	8.7	12.0	60.6	58.2	19.0	16.6
2 or more hours a day	28.9	33.3	8.9	16.8	71.1	55.6	6.7	15.0
*p*-value	n.s.	0.016	n.s.	n.s.	n.s.	n.s.	0.038	n.s.

Percentages were calculated from valid responses; deviations from the total sample size in the column headers were due to non-response. “Burden due to price increases” refers to the perceived strength of financial strain rated on a five-point Likert scale as moderate, strong, or very strong. *p*-values were based on Pearson’s χ^2^ tests for categorical variables and Mann–Whitney U tests for ordinal/continuous variables. ^1^ FAS III (low) refers to the lowest tertile of the FAS III (0–4 points), indicating low material affluence. Abbreviations: FAS, Family Affluence Scale; n.s., not significant.

**Table 4:** Kendall’s tau-b correlations between socioeconomic stressors and mental health outcomes in adolescents.

Mental Health	Stressor	Total Sample	Male	Female	11–14 yrs	15–19 yrs
Depressive symptoms (PHQ-2 score)	Family Affluence	0.014	0.049	–0.017	0.001	0.024
	Burden due to price increases					
	Parental (parent report)	0.166 ***	0.126 ***	0.218 ***	0.151 ***	0.149 ***
	Adolescent (self-report)	0.088 ***	0.037	0.141 ***	0.086 **	0.049
	Adolescent (parent report)	0.038	0.037	0.047	0.040	0.056
Anxiety symptoms (SCARED-GAD score)	Family Affluence	0.011	0.062 *	–0.033	0.006	0.015
	Burden due to price increases					
	Parental (parent report)	0.190 ***	0.194 ***	0.207 ***	0.153 ***	0.213 ***
	Adolescent (self-report)	0.089 ***	0.085 **	0.097 ***	0.070 *	0.087 **
	Adolescent (parent report)	0.070 ***	0.086 **	0.067 *	0.076 **	0.078 **
Emotional/behavioral difficulties (SDQ score)	Family Affluence	–0.032	0.011	–0.070 **	–0.047	–0.016
	Burden due to price increases					
	Parental (parent report)	0.156 ***	0.158 ***	0.160 ***	0.152 ***	0.152 ***
	Adolescent (self-report)	0.041 *	0.032	0.052	0.031	0.039
	Adolescent (parent-report)	0.039	0.046	0.038	0.018	0.067 *

Significance: * *p* < 0.05, ** *p* < 0.01, *** *p* < 0.001; n.s. = non-significant (*p* ≥ 0.05). Effect size interpretation: τ ≈ 0.10 indicates a small effect, τ ≈ 0.20 a typical (medium) effect, and τ ≥ 0.30 a relatively large effect.

**Table 5:** ANOVA models predicting SCARED total scores from combinations of adolescent and parent financial burden perceptions, stratified by age group.

Predictor	11–14 Years ηp^2^ (*p*)	15–19 Years ηp^2^ (*p*)
Self-reported vs. proxy-reported adolescent burden		
Self-reported	0.000 (0.672)	0.001 (0.378)
Proxy-reported	0.000 (0.555)	0.013 (0.002)
Interaction (Self × Proxy)	0.009 (0.008)	0.001 (0.456)
Model *p*-value	<0.001	<0.001
N	779	740
Levene (median)	n.s.	n.s.
Self-reported adolescent burden vs. parental burden		
Self-reported	0.015 (0.001)	0.035 (<0.001)
Parental self	0.001 (0.447)	0.001 (0.302)
Interaction (Self × Parental self)	0.000 (0.580)	0.000 (0.988)
Model *p*-value	<0.001	<0.001
N	783	744
Levene (median)	n.s.	n.s.
Proxy-reported adolescent burden vs. parental burden		
Proxy-reported	0.007 (0.021)	0.003 (0.110)
Parental self	0.004 (0.088)	0.001 (0.317)
Interaction	0.006 (0.025)	0.000 (0.804)
Model *p*-value	0.008	0.006
N	777	739
Levene (median)	n.s.	n.s.

Effect sizes are reported as partial eta squared (ηp^2^) derived from ANOVA models with fixed effects for adolescent burden (self-report), parent-perceived adolescent burden (proxy report), and parental self-burden, including their interaction terms. The dependent variable was the total SCARED score. The models were stratified by age group. n.s. = not significant at *p* ≥ 0.05. Levene’s test was used to evaluate the assumption of homogeneity of variance.

## Data Availability

Data are available from the corresponding author upon reasonable request.

## Abbreviations

The following abbreviations are used in this manuscript:

ANOVA	Analysis of variance
CASMIN	Comparative Analysis of Social Mobility in Industrial Nations
CI	Confidence interval
COP-S	Corona and Psyche South Tyrol
COPSY	Corona and Psyche
COVID-19	Corona virus disease 2019
FAS	Family affluence scale
OR	Odds ratio
GAD	Generalized Anxiety Subscale
PHQ-2	Patient Health Questionnaire-2
SCARED	Screen for Child Anxiety-Related Emotional Disorders
SD	Standard deviation
SDQ	Strengths and Difficulties Questionnaire
SES	Socioeconomic status
